# PTRF/Cavin-1 Deficiency Causes Cardiac Dysfunction Accompanied by Cardiomyocyte Hypertrophy and Cardiac Fibrosis

**DOI:** 10.1371/journal.pone.0162513

**Published:** 2016-09-09

**Authors:** Takuya Taniguchi, Naoki Maruyama, Takehiro Ogata, Takeru Kasahara, Naohiko Nakanishi, Kotaro Miyagawa, Daisuke Naito, Tetsuro Hamaoka, Masahiro Nishi, Satoaki Matoba, Tomomi Ueyama

**Affiliations:** Department of Cardiovascular Medicine, Graduate School of Medical Science, Kyoto Prefectural University of Medicine, Kyoto 602–8566, Japan; University of British Columbia, CANADA

## Abstract

Mutations in the *PTRF/Cavin-1* gene cause congenital generalized lipodystrophy type 4 (CGL4) associated with myopathy. Additionally, long-QT syndrome and fatal cardiac arrhythmia are observed in patients with CGL4 who have homozygous *PTRF*/*Cavin-1* mutations. PTRF/Cavin-1 deficiency shows reductions of caveolae and caveolin-3 (Cav3) protein expression in skeletal muscle, and Cav3 deficiency in the heart causes cardiac hypertrophy with loss of caveolae. However, it remains unknown how loss of PTRF/Cavin-1 affects cardiac morphology and function. Here, we present a characterization of the hearts of *PTRF*/*Cavin-1*-null (*PTRF*^−/−^) mice. Electron microscopy revealed the reduction of caveolae in cardiomyocytes of *PTRF*^−/−^ mice. *PTRF*^−/−^ mice at 16 weeks of age developed a progressive cardiomyopathic phenotype with wall thickening of left ventricles and reduced fractional shortening evaluated by echocardiography. Electrocardiography revealed that *PTRF*^−/−^ mice at 24 weeks of age had low voltages and wide QRS complexes in limb leads. Histological analysis showed cardiomyocyte hypertrophy accompanied by progressive interstitial/perivascular fibrosis. Hypertrophy-related fetal gene expression was also induced in *PTRF*^−/−^ hearts. Western blotting analysis and quantitative RT-PCR revealed that Cav3 expression was suppressed in *PTRF*^−/−^ hearts compared with that in wild-type (WT) ones. ERK1/2 was activated in *PTRF*^−/−^ hearts compared with that in WT ones. These results suggest that loss of PTRF/Cavin-1 protein expression is sufficient to induce a molecular program leading to cardiomyocyte hypertrophy and cardiomyopathy, which is partly attributable to Cav3 reduction in the heart.

## Introduction

Caveolae are flask-shaped invaginations of the plasma membrane characterized by the presence of caveolin proteins [caveolin-1 (Cav1), caveolin-2 (Cav2), and caveolin-3 (Cav3)]. Cav1 is expressed in a variety of cells and tissue types, except for ventricular and skeletal muscle cells, as is Cav2, while Cav3 is primarily expressed in all muscle cell types (cardiac, skeletal, and smooth muscle cells) [1,2]. Cav1 overexpression in cells lacking endogenous caveolin/caveolae, such as lymphocytes and transformed fibroblasts, induces the de novo formation of caveolae [2–4]. The targeted disruption of *Cav1* leads to the loss of caveolae in Cav1-expressing tissues, but their retention in muscle tissues, and ablation of *Cav3* causes the loss of caveolae in striated muscle [1]. Caveolae play an important role in the regulation of many cellular functions, including signal transduction, endocytosis, and lipid regulation [2,5], and caveolins function as scaffolding proteins, recruiting various signaling molecules to caveolae, as well as regulating their activity [1]. *Cav1*-null (*Cav1*^−/−^) mice exhibit hypertrophy and dilatation of both ventricles, pulmonary hypertension, and metabolic disorder [1,2,6]. *Cav3*-null (*Cav3*^−/−^) mice show progressive cardiomyopathy, myopathic changes of the skeletal muscle, and impaired glucose tolerance and insulin resistance [7,8]. Mutations in the *Cav3* gene have been found in patients with hypertrophic cardiomyopathy, long-QT syndrome, and muscular dystrophies [7,9–12]. Thus, caveolins had been recognized as the sole structural component of caveolae necessary and sufficient to regulate caveola biogenesis. However, recent characterizations of polymerase I and transcript release factor (PTRF)/Cavin-1 and subsequently other cavin family members, serum deprivation protein response (SDPR)/Cavin-2 and SDR-related gene product that binds to C kinase (SRBC)/Cavin-3, revealed that they also play critical roles in the biogenesis of caveolae [5,13–16].

PTRF/Cavin-1 can be localized to caveolae and is required for caveola formation [15]. *PTRF*/*Cavin-1*-null (*PTRF*^−/−^) mice have no morphologically detectable caveolae in lung, intestine, and skeletal muscle, with diminished protein expression of all three caveolins, and exhibit glucose intolerance, dyslipidemia, and muscular dystrophy [17]. *PTRF*^−/−^ mice also exhibit a lack of caveolae in the detrusor, impaired contractility, and detrusor hypertrophy [18]. Recent reports have demonstrated elevated pulmonary arterial pressure accompanying hypertrophic remodeling of pulmonary arteries and maintained systemic blood pressure despite arterial dysfunction in *PTRF*^−/−^ mice [19,20]. In humans, homozygous mutations in the *PTRF*/*Cavin-1* gene have been reported to cause muscular dystrophy with lipodystrophy [21]. Furthermore, in patients with congenital generalized lipodystrophy with muscle rippling (CGL4) who have homozygous *PTRF*/*Cavin-1* mutations, long-QT syndrome and fatal cardiac arrhythmia were observed [22,23]. Caveolae are abundantly present in ventricular, atrial, and nodal cells [24], and PTRF/Cavin-1 is expressed in the heart [15,17,25,26]. However, the specific impact of PTRF/Cavin-1 on the molecular mechanisms involved in cardiac hypertrophy and function remains to be determined.

To obtain further insights into caveola function in cardiomyocytes, we utilized *PTRF*^−/−^ mice and found that PTRF/Cavin-1 deficiency induces down-regulated expression of caveola-associated proteins in the heart and cardiac hypertrophy accompanied by fibrosis.

## Materials and Methods

### Materials

Mouse monoclonal anti-Cav1 (Cat. #: 610406, clone name: 2297/Caveolin 1) and anti-Cav3 (Cat. #: 610420, clone name: 26/Caveolin 3) antibodies were from BD Transduction Laboratories. Mouse monoclonal anti-glyceraldehyde-3-phosphate dehydrogenase (GAPDH) antibody (Cat. #: MAB374, clone name: 6C5) was from Merck Millipore. Rabbit polyclonal anti-PTRF/Cavin-1 (Cat. #: ab48824) and anti-SRBC/Cavin-3 (Cat. #: 16250-1-AP) antibodies were from Abcam and Proteintech, respectively. Rabbit polyclonal anti-MURC/Cavin-4 antibody was generated as previously described [27,28]. Rabbit monoclonal anti-phospho-ERK1/2 (pERK, Cat. #: 9101S), rabbit monoclonal anti-ERK (Cat. #: 9102S), rabbit polyclonal anti-phospho-Akt (pAkt, Cat. #: 9271S), rabbit polyclonal anti-Akt (Cat. #: 9272S), rabbit polyclonal anti-phospho-JNK (pJNK, Cat. #: 9251S), rabbit polyclonal anti-JNK (Cat. #: 9252S), mouse monoclonal anti-phospho-p38 (pp38, Cat. #: 9216S, clone name: 28B10), and rabbit polyclonal anti-p38 (Cat. #: 9212) antibodies were from Cell Signaling Technology, Inc. Other materials were obtained from commercial sources.

### Animals

*PTRF*^−/−^ mice were purchased from Jackson Laboratory (stock no. 010502; generated by P. Pilch) [17]. All of the aspects of animal care and experimentation performed in this study were approved by the Institutional Animal Care and Use Committee of Kyoto Prefectural University of Medicine.

### Transmission electron microscopy and quantitation

Twelve-week-old female mouse hearts were fixed with 2% glutaraldehyde in 0.1 M cacodylate buffer, post-fixed with 2% OsO_4_, and stained with uranyl acetate and lead citrate. Microtome sections were examined under an H-7100 transmission electron microscope (HITACHI, Tokyo, Japan) and photographed at a magnification of ×5,000 or ×10,000. Caveolae were identified by their characteristic membrane profiles open at the cell surface. The frequency of caveolae in cardiomyocytes was quantified by measuring both the number of caveolae and the total length of the plasma membrane in multiple electron micrographs obtained from hearts.

### Echocardiography and electrocardiography (ECG)

Echocardiographic and electrocardiographic analyses of mice were performed as described previously [27,28]. After mice had been anesthetized with isoflurane (1.5–20% for maintenance; up to 5% for induction; Abbott Laboratories Pty Ltd.), echocardiography was performed in mice whose heart rate (HR) was between 500/min and 600/min using a Vevo 2100 system (VisualSonics) equipped with a 30-MHz microprobe. For electrocardiography, mice were anesthetized with 2,2,2-tribromoethanol (0.20 mg/g, Sigma-Aldrich) and electrocardiographic recordings were performed using CardiofaxVET ECG-1950 (Nihon Kohden, Tokyo, Japan).

### Histological analysis

Cardiac perfusion-fixation was performed using 4% paraformaldehyde (PFA)/PBS. In brief, after the mice had been sacrificed by cervical dislocation, the thorax was cut to expose the heart and then 4% PFA was manually infused from the apex of the left ventricle. Hearts were cut at the horizontal short-axis plane and heart sections were stained with hematoxylin and eosin (H&E) or Masson’s trichrome. For the measurement of cross-sectional area of cardiomyocytes, heart sections cut at the level of the papillary muscle were stained with H&E, in which regions that included the circular shape of capillaries were selected from the epicardial side of the LV free walls, and ImageJ software (National Institutes of Health) was used as described previously [27–29]. At least 100 cells were measured in each mouse.

### RNA extraction and quantitative reverse transcriptase (RT)-PCR

Total RNA was extracted from hearts using Trizol reagent (Invitrogen) and then treated with DNase I (Qiagen) to remove any residual DNA. Five hundred nanograms of total RNA was converted to cDNA using the High Capacity cDNA Reverse Transcription Kit (Applied Biosystems). Synthesized cDNA was analyzed by kinetic real-time PCR using TaKaRa PCR Thermal Cycler Dice (TAKARA BIO INC., Japan) with Platinum SYBR Green qPCR Super Mix (Invitrogen). Mouse GAPDH was used for normalization because the distribution of the cycle threshold values did not differ significantly between WT and *PTRF*^−/−^ hearts (S1 Fig). The primers used are described in S1 Table.

### Production of polyclonal antibody to SDPR/Cavin-2

Rabbit immunization was conducted by Medical & Biological Laboratories Co., Ltd. (Nagoya, Japan). Synthetic peptides spanning fragments of mouse SDPR/Cavin-2 with C-terminal amidation (CKKSLTPNHQKASSGKS) were conjugated with keyhole limpet hemocyanin (KLH). The KLH-conjugated peptide was mixed sufficiently with Freund’s complete adjuvant to give a suspension, which was injected intradermally into rabbits (female Japanese White). The same quantity of the immunogen, the KLH-conjugated peptide with Freund’s incomplete adjuvant, was injected 7 times, once every week. Then, bleeding was performed to collect the maximum amount of serum. The serum was purified by affinity chromatography against rProteinA (GE Healthcare).

### Western blot analysis

Heart lysates were extracted with a lysis buffer containing 20 mM Tris, pH 7.5, 137 mM NaCl, 1% NP-40, 10% glycerol, and 1× Halt protease and phosphatase inhibitor single-use cocktail, EDTA-free (Pierce, Cat. #: 78443). Heart lysates were electrophoresed in SDS-PAGE and transferred to polyvinylidene difluoride membranes (Millipore). These membranes were subsequently incubated with primary antibodies. Horseradish peroxidase-conjugated antibodies to rabbit IgG and mouse IgG (GE Healthcare) were used as secondary antibodies.

### Statistical analysis

All experiments were performed at least three times. Data are expressed as means ± standard errors and were analyzed using Student’s *t*-test. Deviation from a Mendelian distribution was analyzed using chi-square test. A *P* value of <0.05 was considered significant.

## Results

### *PTRF*^−/−^ hearts show cardiomyocyte hypertrophy associated with fibrosis

When we genotyped offspring from heterozygous *PTRF*^+/−^ parents at 4–6 weeks, 5.7% (4/70) were *PTRF*^−/−^ mice (one female and 3 males) and 24.3% (17/70) were WT mice (7 females and 10 males), indicating that *PTRF*^**−/−**^ mice were born at less than the expected Mendelian frequency (*P*<0.01), which is similar to the finding in a previous report [18]. In addition, when *PTRF*^+/−^ female and *PTRF*^−/−^ male mice were mated, 18.3% (11/60) were *PTRF*^−/−^ mice (9 females and 2 males) and 81.7% (49/60) were *PTRF*^+/−^ mice (21 females and 28 males). This distribution also differs from the expected Mendelian frequency (*P*<0.01). Thus, we hardly obtained *PTRF*^−/−^ male mice. As a consequence, we used *PTRF*^−/−^ female mice in subsequent studies except for electrocardiographic studies. In subsequent studies, strain-, and age-matched *PTRF*^−/−^ mice were compared with WT controls.

**Fig 1:**
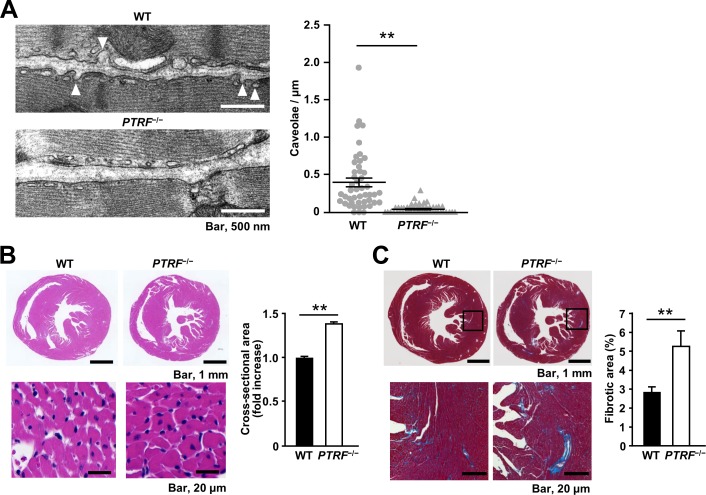
Morphological changes in the heart of *PTRF*^−/−^ mice. (A) Left, representative electron microscopic images of WT and *PTRF*^−/−^ hearts from 12-week-old female mice. Caveolae were identified by their characteristic flask shape and location at the plasma membrane. White arrowheads indicate caveolae. Right, quantification of caveolae per μm of plasma membrane in atrial and ventricular cardiomyocytes of WT and *PTRF*^−/−^ hearts. Multiple electron micrographs were obtained for each heart, and both the number of caveolae and the total length of the plasma membrane were quantified in each image. Caveolae were counted as omega-shaped membrane profiles open at the cell surface. (B) Myocyte cross-sectional area of WT and *PTRF*^−/−^ hearts. Left, representative H&E staining sections of hearts from WT and *PTRF*^−/−^ female mice at 16 weeks of age. Right, bar graph showing cross-sectional area of cardiomyocytes of WT and *PTRF*^−/−^ hearts. (C) Fibrotic area of WT and *PTRF*^−/−^ female hearts. Left, representative Masson’s trichrome staining sections of hearts from WT and *PTRF*^−/−^ female mice at 16 weeks of age. Right, bar graph showing fibrotic area in WT and *PTRF*^−/−^ hearts. **P < 0.01.

Lack or reduction of caveolae has been reported in smooth muscle and skeletal muscle from *PTRF*^−/−^ mice and in skeletal muscle from patients with a homozygous mutation in the *PTRF/Cavin-1* gene [17–19,21]. We examined whether PTRF/Cavin-1 deficiency similarly leads to a reduction of caveolae in the heart. Electron microscopy revealed that the plasma membrane of *PTRF*^−/−^ cardiomyocytes was nearly flat and caveolae that were identified by omega-shaped membrane profiles opening at the cell surface were reduced in *PTRF*^−/−^ cardiomyocytes compared with those in WT cardiomyocytes, although some small vesicles were observed under the plasma membrane of *PTRF*^−/−^ cardiomyocytes (Fig 1A).

At 16 weeks of age, *PTRF*^−/−^ female mice had lower body weight (BW) and shorter tibial length (TL) than WT female mice (Table 1). However, heart-weight-to-BW ratio (HW/BW) and HW-to-TL ratio (HW/TL), which are HW normalized to BW and TL, did not differ between WT and *PTRF*^−/−^ female mice. Echocardiography revealed significant wall thickening of left ventricles and reduced fractional shortening in *PTRF*^−/−^ mice compared with those in WT mice (Table 2, S2 Fig and S1 and S2 Videos). Histological analysis showed that the cross-sectional area of cardiomyocytes in *PTRF*^−/−^ mice was greater than that in WT mice (Fig 1B). *PTRF*^−/−^ mice also had increased interstitial and perivascular fibrosis compared with WT mice (Fig 1C and S3 Fig). Furthermore, 24-week-old *PTRF*^−/−^ mice showed increased fibrosis in the atrium (S4 Fig).

**Table 1:** Morphometric analysis of WT and *PTRF*^−/−^ female mice at 16 weeks of age.

	WT (n = 8)	*PTRF*^−/−^ (n = 6)
BW (g)	24.0±0.6	19.7±0.3**
HW (mg)	123.7±5.4	107.1±3.1*
TL (mm)	16.9±0.2	15.6±0.1**
HW/BW (mg/g)	5.15±0.17	5.44±0.16
HW/TL (mg/mm)	7.32±0.33	6.88±0.19
LW (mg)	142.1±9.9	162.4±4.4
LW/BW (mg/g)	5.96±0.46	8.24±0.13**
LW/TL (mg/mm)	8.38±0.53	10.43±0.26**

BW, body weight; HW, heart weight; TL, tibial length; LW, lung weight. Values are expressed as means ± SEM.

*P < 0.05 and

**P < 0.01 compared with WT mice.

**Table 2:** Echocardiographic analysis of WT and *PTRF*^−/−^ female mice at 16 weeks of age.

	WT (n = 5)	*PTRF*^−/−^ (n = 6)
LVDd (mm)	3.53±0.09	2.99±0.07**
LVDs (mm)	2.33±0.12	2.27±0.09
IVSTd (mm)	0.61±0.04	0.80±0.04**
PWTd (mm)	0.62±0.05	0.87±0.05**
FS (%)	34.3±2.2	24.2±1.5**
EF (%)	63.9±3.0	49.7±2.9**

LVDd, left ventricular dimension at end-diastole; LVDs, left ventricular dimension in systole; IVSTd, interventricular septum thickness at end-diastole; PWTd, left ventricular posterior wall thickness at end-diastole; FS, fractional shortening; EF, ejection fraction. Values are expressed as means ± SEM.

**P < 0.01 compared with WT mice.

**Fig 2:**
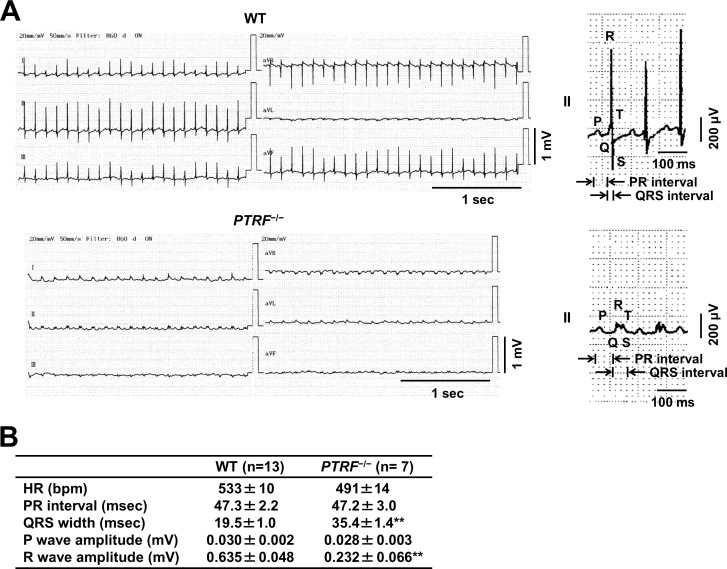
Electrocardiogram of *PTRF*^−/−^ mice. (A) Left, representative ECG of WT and *PTRF*^−/−^ female mice at 24 weeks of age. Right, magnified waveforms of ECG in lead II. (B) ECG parameters in lead II of WT and *PTRF*^−/−^ female mice at 24 weeks of age. HR, heart rate; bpm, beats per minute. Values are expressed as means ± SEM. **P < 0.01 compared with WT mice.

Electrocardiography was performed in WT and *PTRF*^−/−^ mice at the ages of 8, 18, and 24 weeks, although it was not sequentially performed using the same mice. Electrocardiography revealed that, although *PTRF*^−/−^ mice at 8 weeks of age did not show electrocardiographic abnormality (S5 Fig), *PTRF*^−/−^ mice at 18 weeks of age showed tendencies for low voltages and prolonged duration of QRS complexes (S6 Fig). *PTRF*^−/−^ mice at 24 weeks of age had significantly low voltages and prolonged duration of QRS complexes in limb leads compared with WT mice at the same number of weeks of age (Fig 2). Thus, *PTRF*^−/−^ mice showed cardiac contractile dysfunction associated with cardiac fibrosis and electrocardiographic abnormality.

### PTRF/Cavin-1 deficiency stimulates cardiac hypertrophy-related fetal gene expression in the heart

**Fig 3:**
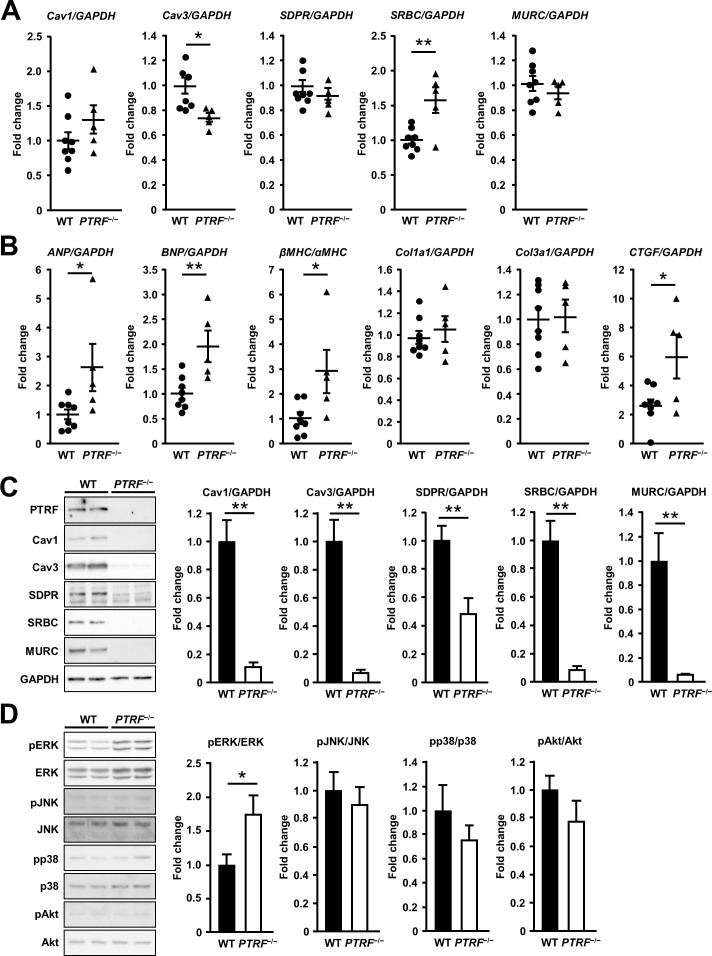
mRNA and protein expression in the heart of *PTRF*^−/−^ mice. (A) mRNA expression of caveolins and cavins in WT and *PTRF*^−/−^ female hearts at 16 weeks of age. (B) mRNA expression of cardiac hypertrophy-related fetal genes and fibrosis-related genes in WT and *PTRF*^−/−^ female hearts at 16 weeks of age. (C) Expression of caveola-associated proteins in WT and *PTRF*^−/−^ female hearts at 16 weeks of age. Left, representative immunoblotting of heart lysates from WT and *PTRF*^−/−^ mice. Right, bar graph showing protein expression of WT and *PTRF*^−/−^ hearts. (D) Phosphorylation levels of MAPKs and Akt in WT and *PTRF*^−/−^ female hearts at 16 weeks of age. Left, representative immunoblotting of heart lysates from WT and *PTRF*^−/−^ mice. Right, bar graph showing phosphorylation levels of MAPKs and Akt in WT and *PTRF*^−/−^ hearts. *P < 0.05 and **P < 0.01.

We then examined the mRNA expression of caveolins and cavins in the hearts of *PTRF*^−/−^ mice at 16 weeks of age. *Cav3* mRNA expression decreased and *SRBC/Cavin-3* mRNA expression increased in *PTRF*^−/−^ hearts compared with those in WT hearts, whereas *Cav1*, *SDPR/Cavin-2*, and *MURC/Cavin-4* mRNA expression did not differ between WT and *PTRF*^−/−^ hearts (Fig 3A). Next, we examined the mRNA expression of cardiac hypertrophy-related fetal gene and fibrosis-related gene. *Atrial natriuretic peptide* (*ANP*) and *brain natriuretic peptide* (*BNP*) mRNA expression was induced in *PTRF*^−/−^ hearts, and the ratio of *β-myosin heavy chain* (*βMHC*) to *α-myosin heavy chain* (*αMHC*) also increased in these hearts (Fig 3B). There was no difference in *collagen type 1 α1* (*Col1a1*) and *collagen type 3 α1* (*Col3a1*) mRNA expression between WT and *PTRF*^−/−^ hearts, while *connective tissue growth factor* (*CTGF*) mRNA expression increased in *PTRF*^−/−^ hearts compared with that in WT hearts. Thus, PTRF/Cavin-1 deficiency stimulates gene expression associated with cardiac hypertrophy and fibrosis.

### PTRF/Cavin-1 deficiency decreases expression of caveola-associated proteins and induces ERK activation in the heart

Previous reports showed that the protein expression of caveolins and cavins was reduced in various tissues, including the heart, of *PTRF*^−/−^ mice [17,26]. In accordance with these reports, the protein expression of Cav-1, Cav-3, SDPR/Cavin-2, SRBC/Cavin-3, and MURC/Cavin-4 markedly decreased in *PTRF*^−/−^ hearts compared with that in WT hearts (Fig 3C). Since Cav3 has been shown to regulate the activity of ERK and Akt in the heart and cardiac hypertrophy [30,31], we examined the phosphorylation of MAPKs and Akt in the heart of 16-week-old *PTRF*^−/−^ mice. ERK1/2 was activated in *PTRF*^−/−^ hearts compared with that in WT ones (Fig 3D). However, JNK, p38, and Akt were not activated in *PTRF*^−/−^ hearts. Thus, PTRF/Cavin-1 deficiency down-regulates caveola-associated protein expression and induces ERK1/2 activation in the heart.

## Discussion

Previous reports have described that *PTRF*^−/−^ mice exhibit loss or reduction of caveolae in endothelium, epithelium, smooth muscle, and skeletal muscle [17–20]. Although PTRF/Cavin-1 protein expression in the heart, as well as other tissues, is absent in *PTRF*^−/−^ mice [17,25,26], it has not been reported whether caveolae are decreased in cardiomyocytes. Here, we demonstrated that caveolae were markedly decreased in *PTRF*^−/−^ cardiomyocytes. Cav3 has been recognized as the sole determinant of caveola formation in cardiomyocytes [31,32]. However, our finding indicates that PTRF/Cavin-1 is also essential for caveola formation in cardiomyocytes and, taken together with previous information [17–20], suggests that PTRF/Cavin-1 governs caveola formation in PTRF/Cavin-1-expressing cells.

Compared with WT mice, *PTRF*^−/−^ mice showed increased cardiomyocyte size evaluated by measuring cross-sectional area of cardiomyocytes and wall thickening of left ventricles assessed by echocardiography, suggesting that increased cardiomyocyte size is attributable to wall thickening of left ventricles in *PTRF*^−/−^ heart. However, morphometric analysis revealed that HW/BW and HW/TL do not differ between WT and *PTRF*^−/−^ mice. These findings also suggest that increased cardiomyocyte size in the *PTRF*^−/−^ heart might be insufficient to increase HW/BW and HW/TL. Considering the findings that increased interstitial and perivascular fibrosis and various sizes of cardiomyocytes were observed in the *PTRF*^−/−^ heart (S3 Fig), it is possible that cardiomyocyte death is also induced in the *PTRF*^−/−^ heart, which might be responsible for the lack of change in HW/BW and HW/TL, despite the presence of cardiomyocyte hypertrophy.

In skeletal muscle and fibroblasts of patients with homozygous mutations in the *PTRF/Cavin-1* gene, reduction and mislocalization of caveolin family proteins have been reported [21,22]. We demonstrated that the mRNA expression of *Cav1*, *SDPR/Cavin-2*, and *MURC/Cavin-4* did not differ between *PTRF*^−/−^ and WT hearts, and that *Cav3* mRNA expression modestly decreased in *PTRF*^−/−^ hearts compared with that in WT ones, while *SRBC/Cavin-3* mRNA expression increased in *PTRF*^−/−^ hearts compared with that in WT ones. On the other hand, the protein levels of Cav1, Cav3, SDPR/Cavin-2, SRBC/Cavin-3, and MURC/Cavin-4 were markedly diminished in *PTRF*^−/−^ hearts compared with those in WT ones, which is consistent with a previous study [26]. In mice, heart failure has been shown to decrease *Cav3* mRNA expression accompanying a concordant change in its protein expression [33], suggesting that decreased *Cav3* mRNA in the *PTRF*^−/−^ heart is, in part, a secondary change attributable to cardiac dysfunction in *PTRF*^−/−^ mice. In addition, disproportionate reductions in Cav1, Cav3, SDPR/Cavin-2, SRBC/Cavin-3, and MURC/Cavin-4 protein compared with their mRNA expression in the *PTRF*^−/−^ heart suggest post-translational regulation, which appears to result from their improper localization due to loss of caveolae in the *PTRF*^−/−^ heart. Thus, PTRF/Cavin-1 is required to stabilize their proteins in caveolae of cardiomyocytes and non-cardiomyocytes in the heart. PTRF/Cavin-1 and SDPR/Cavin-2 show a broad tissue distribution including in cardiomyocytes [25,28,34], while MURC/Cavin-4 is exclusively expressed in cardiomyocytes, skeletal muscle, and smooth muscle [25,28,35]. The present study also shows that, even though *SDPR*/*Cavin-2*, *SRBC*/*Cavin-3*, and *MURC*/*Cavin-4* mRNA are expressed in the *PTRF*^−/−^ heart, cavin family genes such as *SDPR*/*Cavin-2*, *SRBC*/*Cavin-3*, and *MURC*/*Cavin-4* are incapable of compensating for *PTRF*/*Cavin-1* deficiency, suggesting that there is no functional genetic redundancy of *SDPR*/*Cavin-2*, *SRBC*/*Cavin-3*, and *MURC*/*Cavin-4* to compensate for *PTRF*/*Cavin-1* deficiency in cardiomyocytes.

We presented electrocardiograms recorded in *PTRF*^−/−^ mice at the ages of 8, 18, 24 weeks. Although the electrocardiogram recorded in *PTRF*^−/−^ mice at 8 weeks of age did not differ from that in WT at 8 weeks of age, *PTRF*^−/−^ mice at 24 weeks of age showed prolonged duration and low voltages of QRS complexes in limb leads. In humans, prolonged QRS duration usually indicates the presence of changes in the myocardium due to underlying heart disease and is often associated with LV systolic dysfunction in patients with heart failure [36,37]. Therefore, it is possible that systolic dysfunction accompanied by cardiac fibrosis is partly responsible for the ECG abnormalities in the *PTRF*^−/−^ heart. However, it remains unknown whether these ECG abnormalities observed in *PTRF*^−/−^ mice are directly linked with mechanisms underlying arrhythmogenesis in patients with homozygous *PTRF*/*Cavin-1* mutations [22,23]. It has been reported that there are differences in heart size, body mass, and oxygen consumption between mouse and human, which are responsible for the differences in the mouse and human action potential duration [38]. In addition, the ionic currents determining repolarization time in adult mice have been shown to be different from those in humans [39]. For these reasons, although mouse models of human arrhythmia disorders show important phenotypic features, they do not always completely demonstrate a spectrum of the characteristic human pathologies. In patients with homozygous *PTRF*/*Cavin-1* mutations, long-QT syndrome was observed [22]. Heterozygous mutations in *Cav3* have also been reported to be associated with long-QT syndrome (LQT9) [11,40,41], although a report has described no association between long-QT syndrome and *Cav3* mutations [42]. In addition, some of the caveola-localized ion channels have been identified as being associated with susceptibility to heritable arrhythmia syndrome [43]. A number of ion channels such as L-type Ca^2+^ channels (Ca_v_1.2), Na^+^ channels (Na_v_1.5), pacemaker channels (HCN4), and Na^+^/Ca^2+^ exchanger (NCX1) have been found to localize at caveolae in cardiomyocytes, and Cav3 has been shown to be associated with them [2,24,44]. Because caveolae and Cav-3 protein expression are markedly reduced in the *PTRF*^−/−^ heart, it is also possible that mislocalization and/or altered expression of the caveola-localized ion channels in cardiomyocytes are attributable to ECG abnormalities in the *PTRF*^−/−^ heart. Further studies are needed to elucidate the role of PTRF/Cavin-1 in the localization and expression of ion channels in cardiomyocytes.

*Cav1*^−/−^ mice have been shown to develop cardiac hypertrophy with decreased systolic function [45,46], even though Cav1 is expressed in atrial cardiomyocytes and endothelial cells but not in ventricular cardiomyocytes in the mouse heart [47,48]. *Cav3*^−/−^ mice also develop cardiomyopathy with ERK activation in the heart [31], and *Cav1* and *Cav3* double-knockout mice exhibit a severe cardiomyopathic phenotype compared with *Cav1*^−/−^ mice and *Cav3*^−/−^ mice [49]. We revealed that *PTRF*^−/−^ mice exhibit cardiac dysfunction and ERK activation, which are accompanied by Cav1 and Cav3 down-regulation in the heart. Considering the protein levels of Cav1 and Cav3 in the *PTRF*^−/−^ heart and the phenotype of *Cav1* and *Cav3* double-knockout mice, cardiac phenotypes in *PTRF*^−/−^ mice are partly attributable to diminished protein expression of Cav1 and Cav3 in cardiomyocytes and non-cardiomyocytes. We have reported that *MURC*^−/−^ mice display attenuation of α1-adrenergic stimulation-induced ERK activation and cardiac hypertrophy [27]. *PTRF*^−/−^ hearts show ERK activation and the reduction of MURC/Cavin-4 protein. Unlike *PTRF*^−/−^ mice, *MURC*^−/−^ mice show that Cav3 is localized at the plasma membrane of cardiomyocytes [27]. Because Cav1 and Cav3 have been shown to function as negative regulators of the ERK cascade [1], the differences in cardiac phenotypes between *PTRF*^−/−^ and *MURC*^−/−^ mice are probably caused by changes in the protein expression and localization of Cav1 and Cav3 in the heart. SDPR/Cavin-2 has also been reported to regulate caveolar morphology [14,26]. Because caveolins and cavins form complexes [34], compositional changes of the complex probably affect the morphology and function of caveolae, thereby modulating the function of cardiomyocytes.

## Conclusions

In the present study, we showed that PTRF/Cavin-1 deficiency causes loss of caveolae in cardiomyocytes, impaired cardiac function accompanying cardiomyocyte hypertrophy and interstitial fibrosis, reductions in Cav-1, Cav-3, SDPR/Cavin-2, SRBC/Cavin-3, and MURC/Cavin-4, and the activation of ERK in the heart. These findings provide novel insight into the role of PTRF/Cavin-1 in the heart.

## Supporting Information

S1 Fig Distribution of qPCR cycle threshold values for the candidate reference genes in WT and *PTRF*^−/−^ hearts. The expression of *GAPDH*, *β-actin*, and *eukaryotic translation initiation factor EIF35S* (*TIF*) candidate internal control genes in the heart of WT and *PTRF*^−/−^ mice at the age of 16 weeks is presented as box and whisker plots. A circle represents an outlier. *P < 0.05. NS, not significant.
(TIF)

S2 Fig Echocardiographic images in WT and *PTRF*^−/−^ mice. Representative echocardiographic images in WT and *PTRF*^−/−^ female mice at 16 weeks of age.
(TIF)

S3 Fig Morphological changes in the ventricle of *PTRF*^−/−^ mice. Representative Masson’s trichrome staining sections of ventricles from WT and *PTRF*^−/−^ female mice at 16 weeks of age. These are other magnified parts of pictures presented in Fig 1C.
(TIF)

S4 Fig Morphological changes in the atrium of *PTRF*^−/−^ mice. Upper, representative H&E staining sections of atria from WT and *PTRF*^−/−^ female mice at 24 weeks of age. Lower, representative Masson’s trichrome staining sections of atria from WT and *PTRF*^−/−^ female mice at 24 weeks of age.
(TIF)

S5 Fig Electrocardiogram of *PTRF*^−/−^ mice at 8 weeks of age. Left, representative ECG of WT and *PTRF*^−/−^ female mice at 8 weeks of age. Right, magnified waveforms of ECG in lead II.
(TIF)

S6 Fig Electrocardiogram of *PTRF*^−/−^ mice at 18 weeks of age. Left, representative ECG of WT and *PTRF*^−/−^ female mice at 18 weeks of age. Right, magnified waveforms of ECG in lead II.
(TIF)

S7 Fig Uncropped blots in main figures. Full immunoblot images with the corresponding figure and panel numbers are shown in Fig 3C.
(TIF)

S8 Fig Uncropped blots in main figures. Full immunoblot images with the corresponding figure and panel numbers are shown in Fig 3D.
(TIF)

S1 Table Primer sequences for real-time PCR. (DOCX)

S1 Video Echocardiogram of WT mice. Representative echocardiogram in WT female mice at 16 weeks of age.
(AVI)

S2 Video Echocardiogram of *PTRF*^−/−^ mice. Representative echocardiogram in *PTRF*^−/−^ female mice at 16 weeks of age.
(AVI)
