# Supplementary figures and images for: PTRF/Cavin-1 Deficiency Causes Cardiac Dysfunction Accompanied by Cardiomyocyte Hypertrophy and Cardiac Fibrosis
Source: PLoS One. 2016 Sep 9;11(9):e0162513. doi: 10.1371/journal.pone.0162513 (PMC5017623; doi:10.1371/journal.pone.0162513)

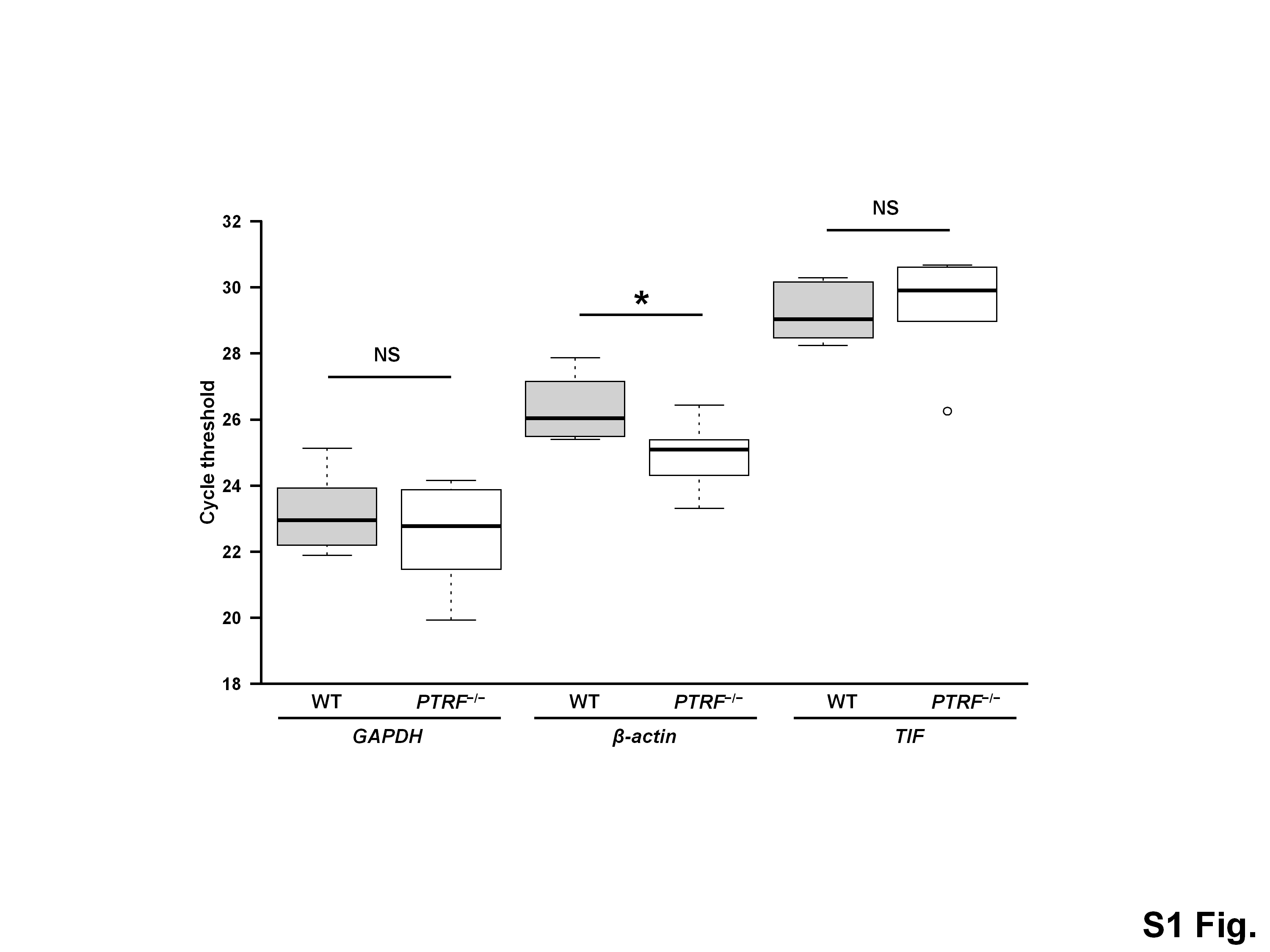

Supplement: S1 Fig — The expression of GAPDH, β-actin, and eukaryotic translation initiation factor EIF35S (TIF) candidate internal control genes in the heart of WT and PTRF−/− mice at the age of 16 weeks is presented as box and whisker plots. A circle represents an outlier. *P < 0.05. NS, not significant. (TIF) [file pone.0162513.s001.TIF]

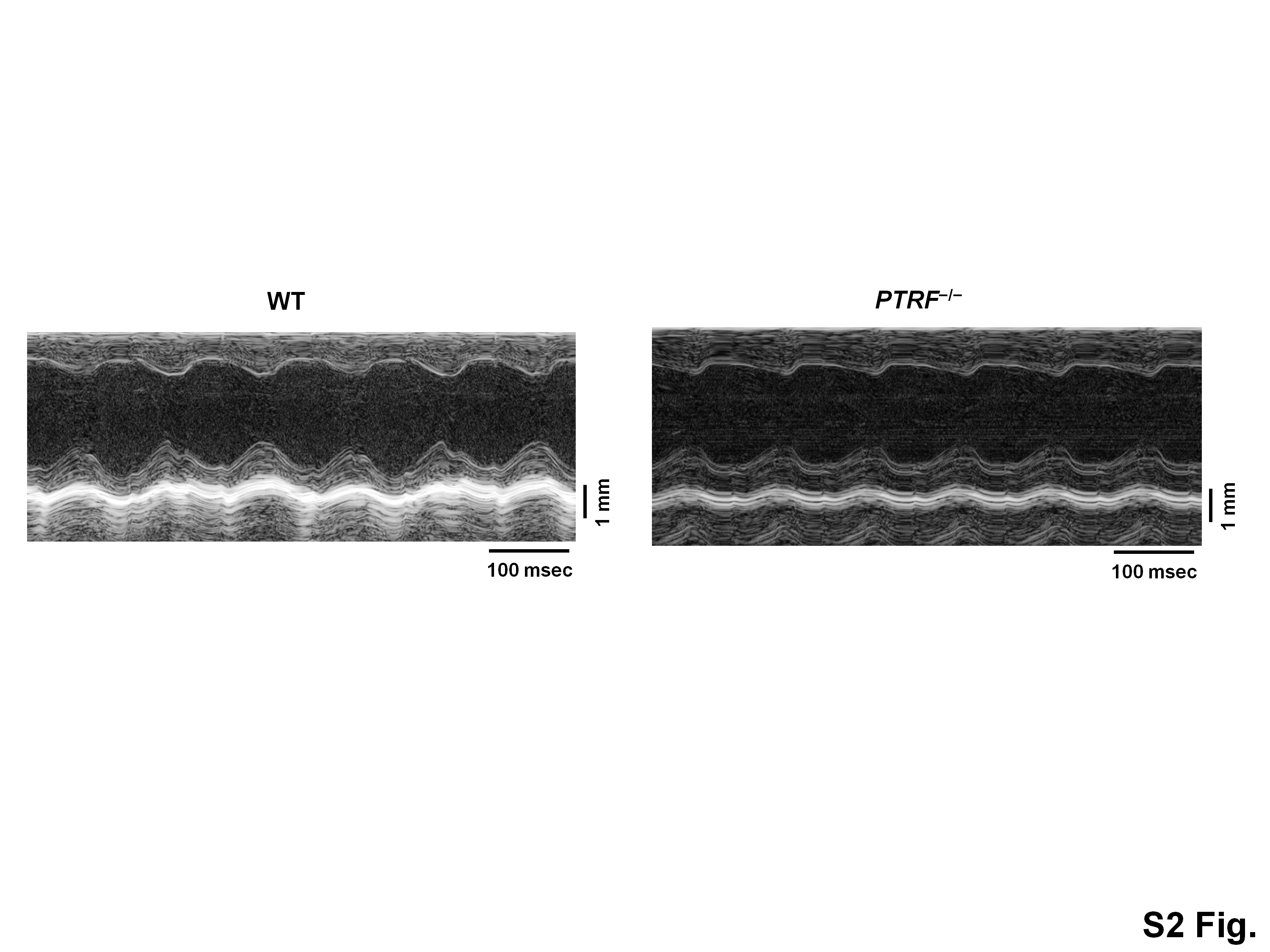

Supplement: S2 Fig — Representative echocardiographic images in WT and PTRF−/− female mice at 16 weeks of age. (TIF) [file pone.0162513.s002.TIF]

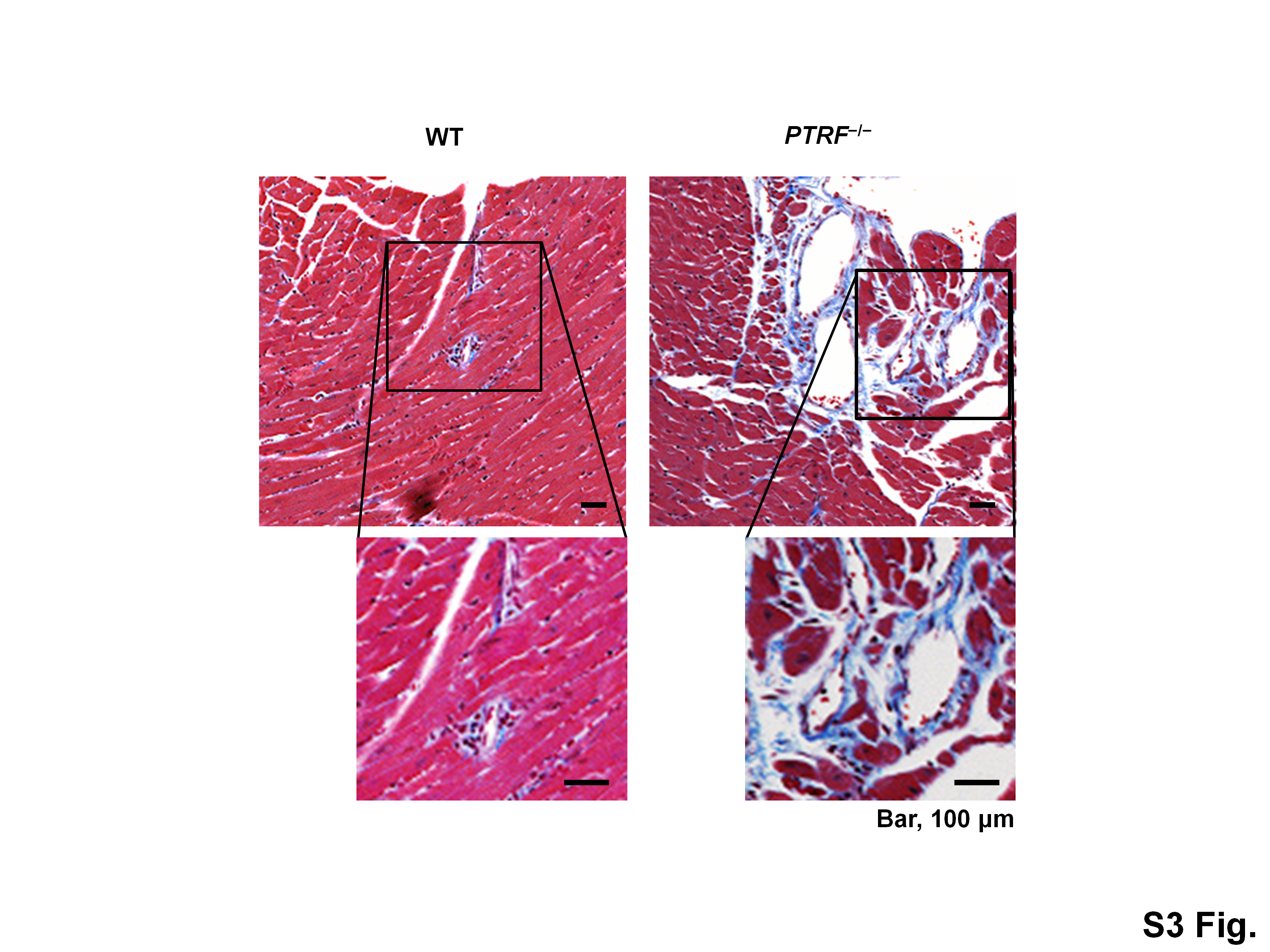

Supplement: S3 Fig — Representative Masson’s trichrome staining sections of ventricles from WT and PTRF−/− female mice at 16 weeks of age. These are other magnified parts of pictures presented in Fig 1C. (TIF) [file pone.0162513.s003.TIF]

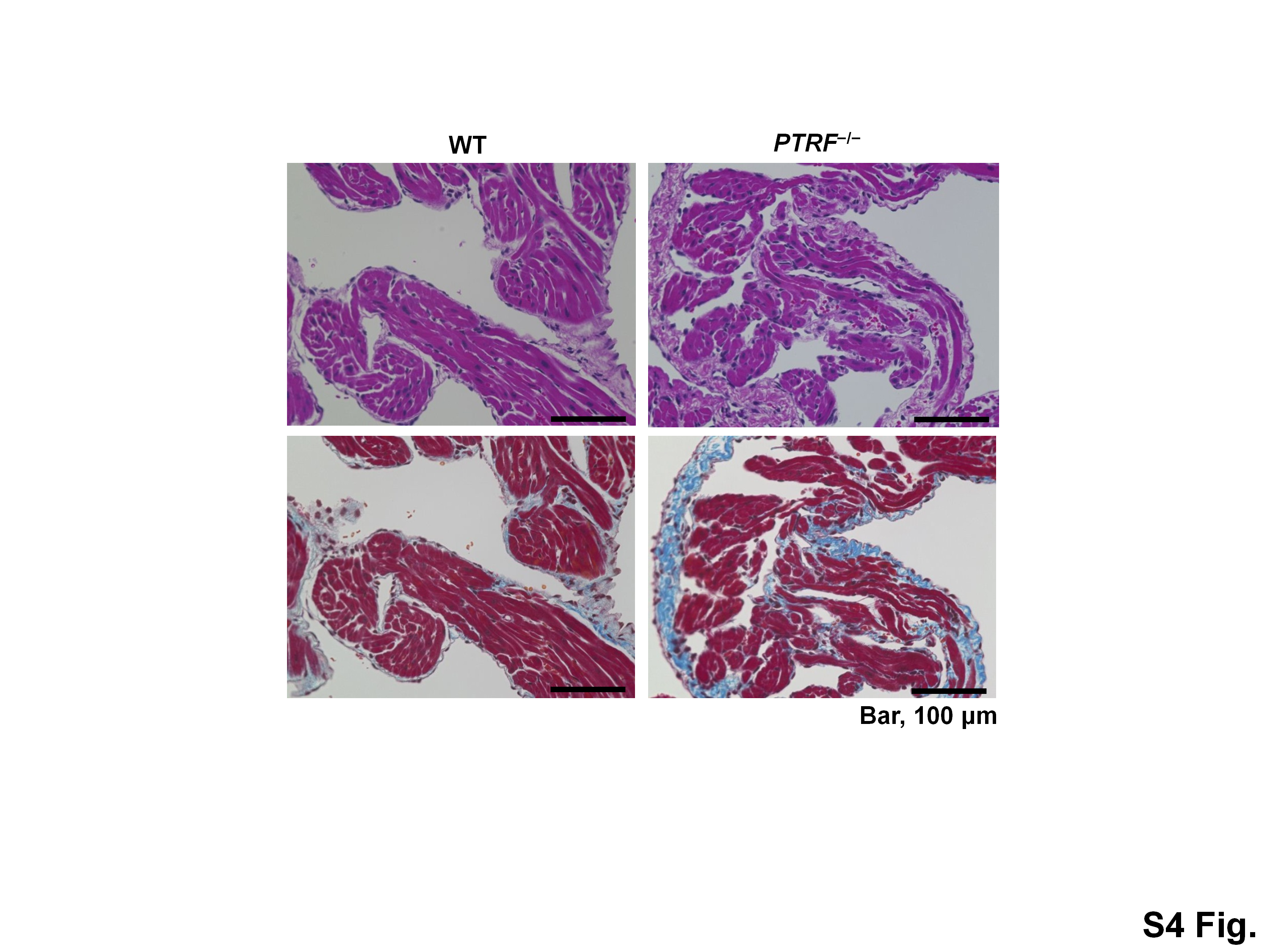

Supplement: S4 Fig — Upper, representative H&E staining sections of atria from WT and PTRF−/− female mice at 24 weeks of age. Lower, representative Masson’s trichrome staining sections of atria from WT and PTRF−/− female mice at 24 weeks of age. (TIF) [file pone.0162513.s004.TIF]

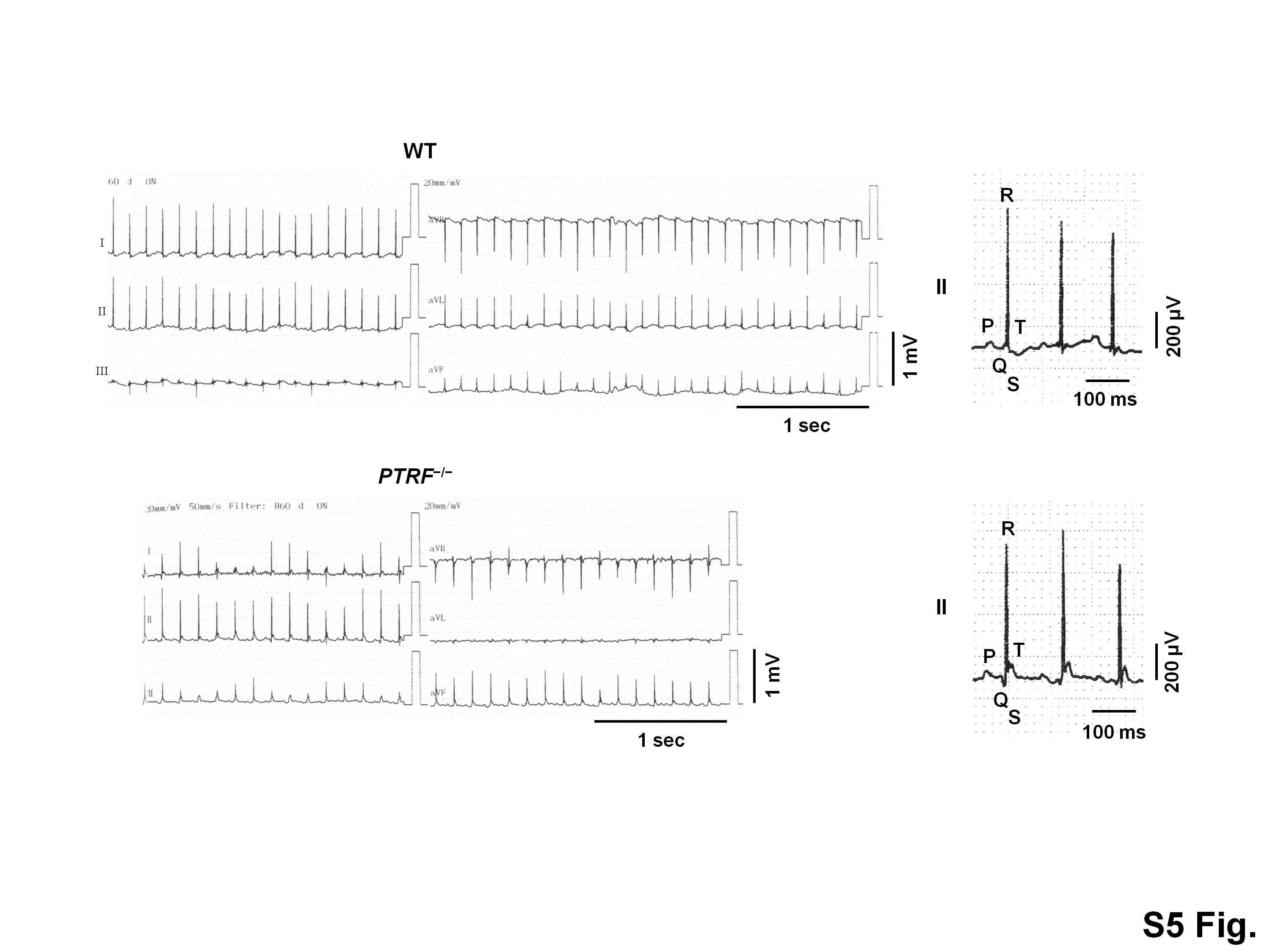

Supplement: S5 Fig — Left, representative ECG of WT and PTRF−/− female mice at 8 weeks of age. Right, magnified waveforms of ECG in lead II. (TIF) [file pone.0162513.s005.TIF]

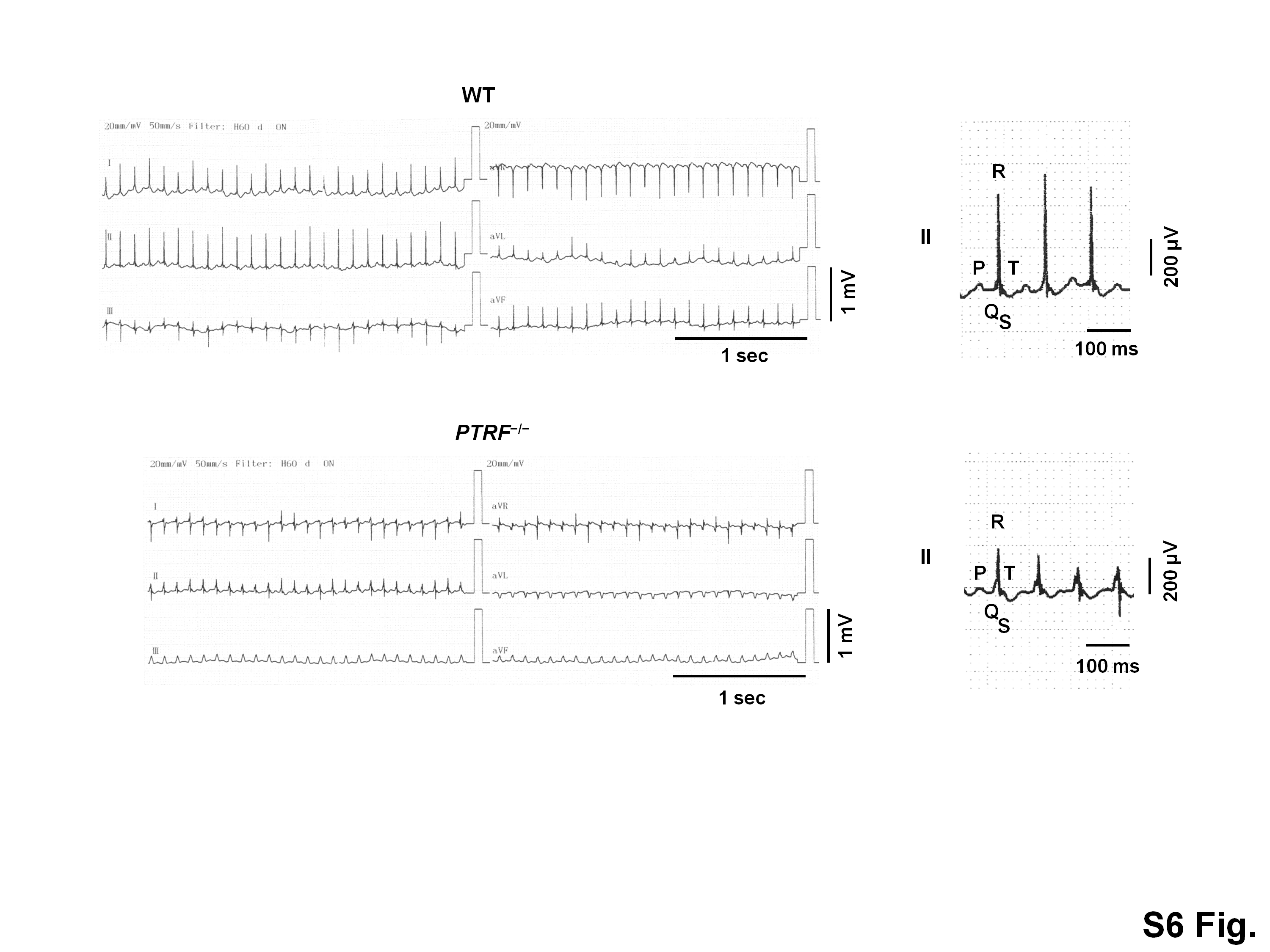

Supplement: S6 Fig — Left, representative ECG of WT and PTRF−/− female mice at 18 weeks of age. Right, magnified waveforms of ECG in lead II. (TIF) [file pone.0162513.s006.TIF]

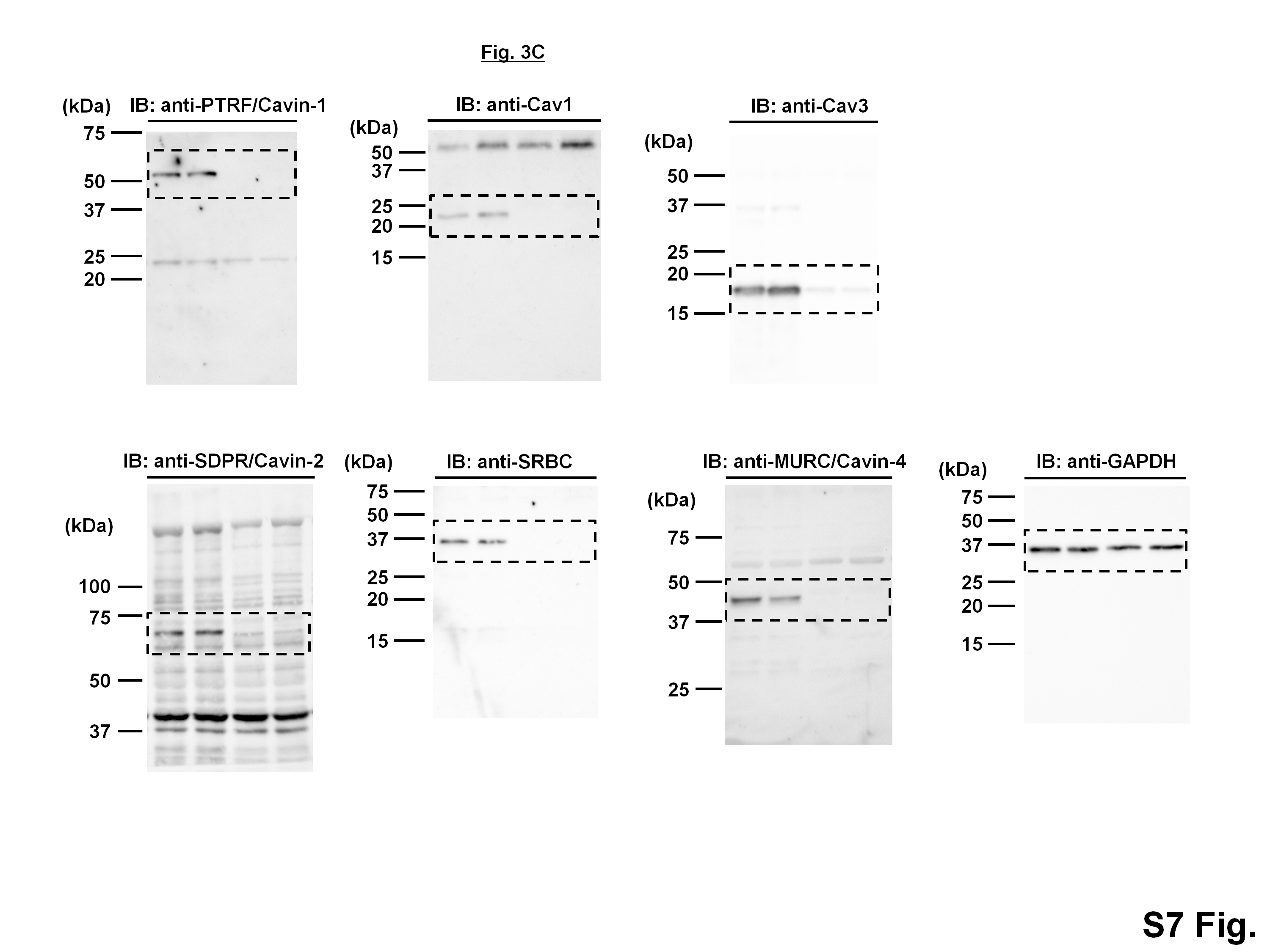

Supplement: S7 Fig — Full immunoblot images with the corresponding figure and panel numbers are shown in Fig 3C. (TIF) [file pone.0162513.s007.TIF]

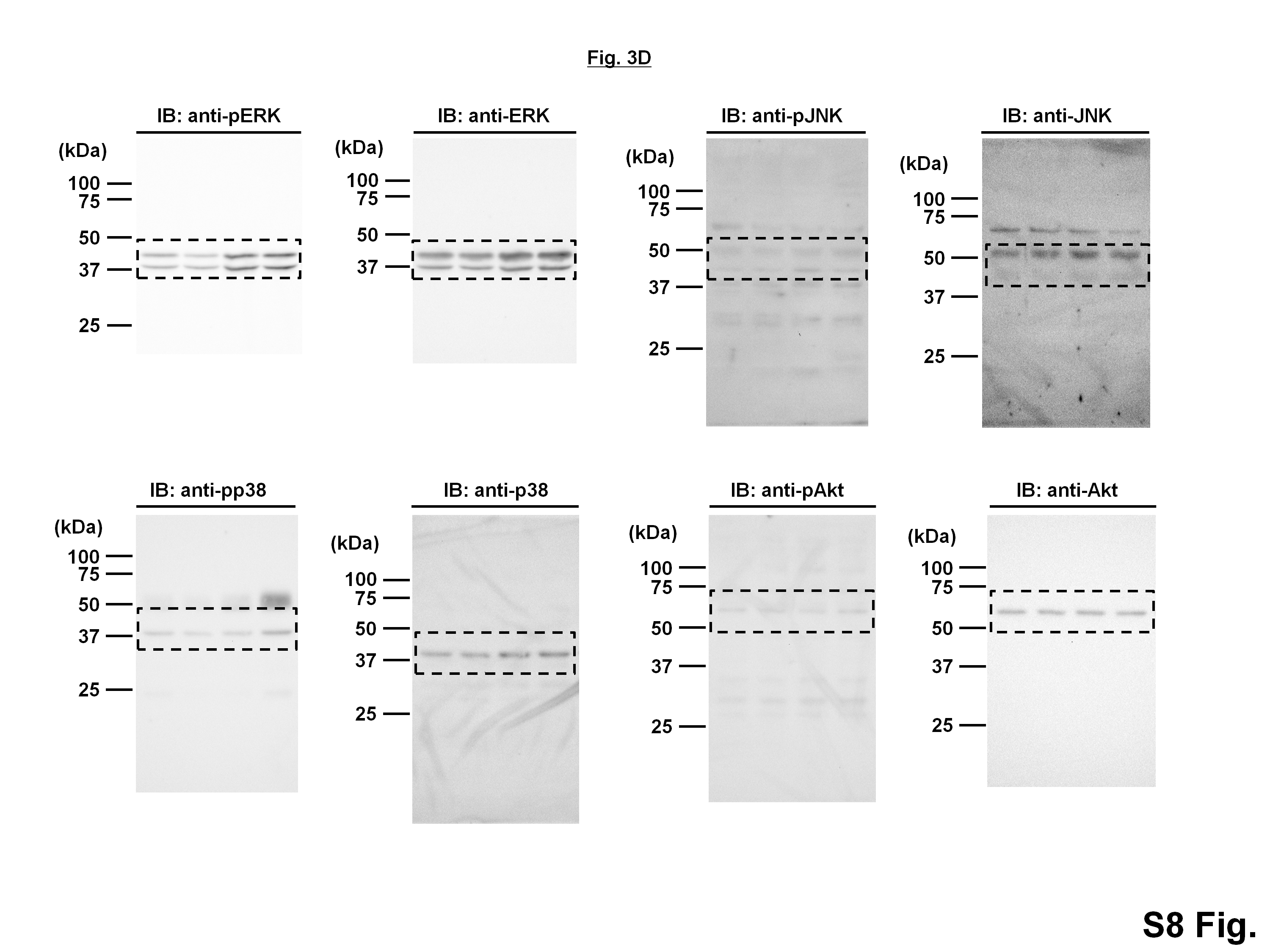

Supplement: S8 Fig — Full immunoblot images with the corresponding figure and panel numbers are shown in Fig 3D. (TIF) [file pone.0162513.s008.TIF]
