# Supplementary material for: PTRF/Cavin-1 Deficiency Causes Cardiac Dysfunction Accompanied by Cardiomyocyte Hypertrophy and Cardiac Fibrosis
Source: PLoS One. 2016 Sep 9;11(9):e0162513. doi: 10.1371/journal.pone.0162513 (PMC5017623; doi:10.1371/journal.pone.0162513)
Supplement: S1 Table — (DOCX) [file pone.0162513.s009.docx]

S1 Table. Primer sequences for real-time PCR

| gene | forward primer | reverse primer |
| --- | --- | --- |
| *Cav1* | CGGGAACAGGGCAACATCTA | TGTGTCCCTTCTTTCTGC |
| *Cav3* | ACGGTGTATGGAAGGTGAGC | GGAGATACAGGCGAACAGGA |
| *SDPR* | ATGAGGAAGCCCTGGAAGAT | CCCAGATGATGCTTTCTGGT |
| *SRBC* | TGAAGAGGAGCCAGTGGAGT | GCTGTGCTGCTTTACGACTG |
| *MURC* | ACAGTCACACAGCAATACGGGCTA | TTCTCGGGCAGGCTTCTGTCTTTA |
| *ANP* | AACCTGCTAGACCACCTGGA | TGCTTTTCAAGAGGGCAGAT |
| *BNP* | CTGAAGGTGCTGTCCCAGAT | CCTTGGTCCTTCAAGAGCTG |
| *αMHC* | GAGGACCAGGCCAATGAGTA | GCTGGGTGTAGGAGAGCTTG |
| *βMHC* | GGAGCTCACCTACCAGACAGA | CTCAGGGCTTCACAGGCATCC |
| *Col1a1* | TGCACCACCAACTGCTTAG | GGATGCAGGGATGATGTTC |
| *Col3a1* | CCCAACCCAGAGATCCCAT | GAAGCACAGGAGCAGGTGTAGA |
| *CTGF* | CAAAGCAGCTGCAAATACCA | GGCCAAATGTGTCTTCCAGT |
| *GAPDH* | TTGTGATGGGTGTGAACCACGAGA | CATGAGCCCTTCCACAATGCCAAA |
| *β-actin* | AGCCATGTACGTAGCCATCC | CTCTCAGCTGTGGTGGTGAA |
| *TIF* | CTGAGGATGTGCTGTCTGGGAA | CCTTTGCCTCCACTTCGGTC |

*Cav1, caveolin-1; Cav3, caveolin-3; SDPR, SDPR/Cavin-2; SRBC, SRBC/Cavin-3; MURC, MURC/Cavin-4; ANP, atrial natriuretic peptide; BNP, brain natriuretic peptide; αMHC, α-myosin heavy chain; βMHC, β-myosin heavy chain; Col1a1, collagen type 1 α1; Col3a1, collagen type 3 α1; CTGF, connective tissue growth factor*; *TIF, eukaryotic translation initiation factor EIF35S*.
